# Pyrolytic Lignin: A Promising Precursor for the Green Synthesis of Fluorescent Carbon Nanoparticles

**DOI:** 10.1021/acsomega.4c09764

**Published:** 2025-03-12

**Authors:** Rosinaldo Rabelo Aparicio, Gabriel Goetten de Lima, Gisele Eliane Perissutti, Maiara de Jesus Bassi, Joslaine Jacumazo, Marco Antônio Schiavon, Lucimara Stolz Roman, Graciela Ines Bolzon de Muniz, Washington Luiz Esteves Magalhães, Pedro Henrique Gonzalez de Cademartori

**Affiliations:** † Materials Science and Engineering Program (PIPE), Federal University of Paraná, Polytechnic Center, Curitiba 81531-990, Brazil; ‡ Federal Institute Catarinense, São Francisco do Sul 89240-000, Brazil; § PRISM Research Institute, Technological University of the Shannon: Midlands Midwest, Athlone N37 HD68, Ireland; ∥ Embrapa Florestas, Colombo 83411-000, Brazil; ⊥ Nanostructured Devices Laboratory at Physics Department, Federal University of Paraná, Curitiba 81531-980, Brazil; # Pharmaceutical Sciences Graduate Program, Federal University of Paraná (UFPR), Curitiba 81531-990, Brazil; ∇ Materials Chemistry Research Group, Department of Natural Sciences, Federal University of São João del-Rei, São João del-Rei 36301-160, Brazil; ○ Forestry Engineering Graduate Program (PPGEF), Federal University of Paraná, Curitiba 80210-170, Brazil

## Abstract

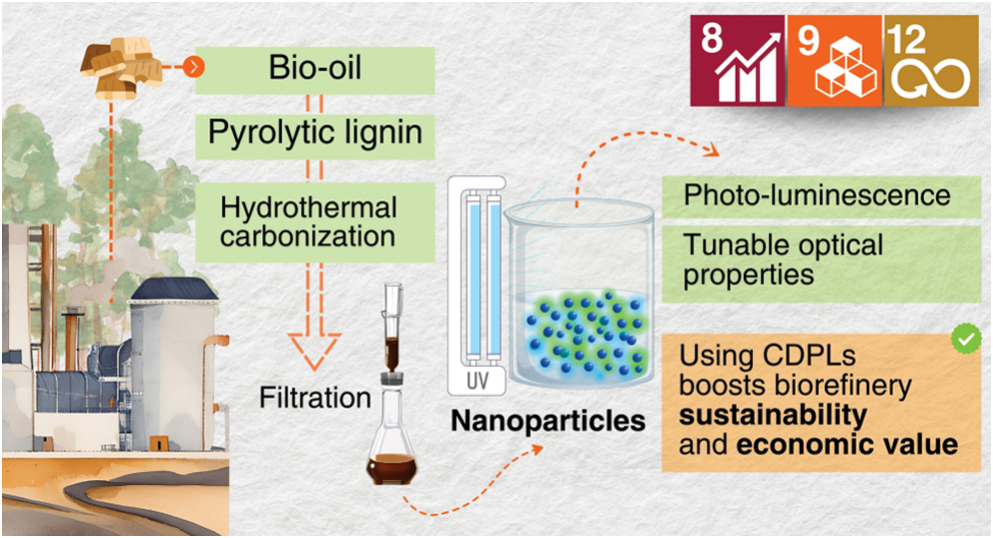

This study presents a simple and cost-effective approach for synthesizing pyrolytic lignin-based carbon dots (CDPLs) via controlled thermal pyrolysis in water. To the best of our knowledge, this is the first time that pyrolytic lignin has been used as a precursor for carbon dots. The one-pot method produced blue/green fluorescent CDPLs with an average size of 34 nm and a negative surface charge of −10.4 mV. The characterization techniques revealed the optical properties and chemical composition of CDPLs, with a fluorescence quantum yield of 7.9%, which is comparable to those of lignin-derived carbon dots. The decay lifetime of CDPLs was in the nanosecond range, which is typical for carbon dots. This study demonstrates the potential of using pyrolytic lignin, a lignocellulosic byproduct, to produce carbon dots through a simple and reproducible method, contributing to the development of sustainable carbon-based nanomaterials.

## 1 Introduction

Carbon dots (CDs) represent a promising class of fluorescent carbon nanoparticles that have attracted increasing interest since their discovery in 2004.^1,2^ Compared with semiconductor quantum dots and organic dyes, carbon dots exhibit remarkable properties such as tunable photoluminescence, chemical inertness, ease of production, low toxicity, and good biocompatibility.^1−5^ These advantageous characteristics give carbon dots broad potential for applications in several fields, including chemical sensing, bioimaging, photocatalysis, and wastewater treatment.^2,3^

The selection of carbon-rich raw materials is crucial for the efficient production of carbon dots.^6^ In this context, lignocellulosic biomass stands out as a sustainable and economical alternative due to its high carbon content and diversity of organic constituents.^7^ Current research has focused intensely on using this biomass as a primary carbon source for the synthesis of carbon dots, with the aim of achieving significant cost reductions and performance improvements.^5,8^

Lignin, a complex aromatic biopolymer, is one of the most abundant natural polymers on earth and a major byproduct of the pulp and paper industry.^9,10^ In recent years, there has been growing interest in valorizing lignin for high-value applications, including the synthesis of nanomaterials^11,12^ and promising raw material for producing carbon dots, facilitating the production of carbon dots with tunable fluorescence emission capacity.^13−15^ Among the various types of lignin, pyrolytic lignin has emerged as a promising precursor for carbon-based nanomaterials.

Pyrolytic lignin is derived from the fast pyrolysis of lignocellulosic biomass, a process that rapidly heats biomass in the absence of oxygen to produce bio-oil.^16^ Common feedstocks for pyrolytic lignin production include wood (e.g., pine, eucalyptus) and agricultural residues (e.g., corn stover, wheat straw).^17^ The choice of biomass source can significantly influence the chemical composition and properties of the resulting pyrolytic lignin.^16^

The chemical composition of pyrolytic lignin exhibits a complex and heterogeneous nature, comprising a diverse array of phenolic compounds and their derivatives.^18^ This intricate mixture includes several key components: phenolic monomers (e.g., guaiacol, syringe, and their alkylated variants), oligomeric phenolic compounds (such as dimers, trimers, and larger lignin fragments), carboxylic acids (notably acetic and formic acids), aldehydes and ketones (including vanillin and acetovanillone), and aliphatic compounds (encompassing short-chain alcohols and hydrocarbons).^19−21^ This diverse chemical profile contributes to the unique properties and potential applications of pyrolytic lignin in various fields, including the synthesis of carbon dots.

Pyrolytic lignin offers several advantages for green nanomaterial synthesis. As a biomass processing byproduct, it provides a sustainable alternative to fossil-based precursors, supporting circular economy principles.^16^ Offers several advantages over other lignin sources, including higher carbon content, lower molecular weight, and increased reactivity due to its partially depolymerized structure.^16,22^ Its carbon-rich aromatic structure enhances the carbon dot synthesis efficiency. The abundant oxygen-containing groups in pyrolytic lignin contribute to carbon dot surface functionalization without additional treatments. Recent advances in carbonized polymer dots (CPDs) have demonstrated the importance of core and shell engineering in tailoring their properties. Wang et al. provide a comprehensive review of CPD synthesis, characterization, and applications, emphasizing the role of structural control in enhancing performance.^23^ Additionally, pyrolytic lignin’s partially depolymerized nature increases reactivity, allowing for milder synthesis conditions and more energy-efficient processes than other lignin sources.

Using pyrolytic lignin for sustainable nanomaterial synthesis aligns with green chemistry and circular economy principles. As nanotechnology advances, the demand for ecofriendly precursors that reduce environmental impact and valorize waste is growing. Pyrolytic lignin, a bio-oil byproduct, could integrate carbon dot synthesis into biorefinery processes, improving the resource efficiency.

Recent reviews suggest that pyrolytic lignin still needs to be explored for carbon dot synthesis despite its promising properties as a carbon-rich feedstock.^24,25^ This gap presents an opportunity for innovation in sustainable nanomaterials.

Our study introduces pyrolytic lignin as a novel raw material for carbon dot synthesis via hydrothermal carbonization. We thoroughly characterized the resulting materials through spectroscopic analysis, particle size measurement, ζ-potential, and morphology studies. This approach aims to validate pyrolytic lignin’s potential as a carbon source for producing carbon dots, adhering to bioeconomy and circular economy principles. By investigating this new precursor, we seek to advance sustainable nanomaterial synthesis and expand lignin valorization possibilities.

## 2 Materials and Methods

### 2.1 Synthesis of Pyrolytic Lignin

The pyrolytic lignin used in this study was extracted from bio-oil generated through fast pyrolysis of dry wood biomass from sustainably managed eucalyptus plantations in central-western Brazil, supplied by Suzano, a large forestry company. The extraction employed a water fractionation process. At room temperature, the process began by adding a 1:50 ratio of bio-oil to distilled water, followed by mechanical agitation at different speeds for specific durations (400 rpm for 1 min, 800 rpm for 2 min, and 1200 rpm for 5 min). After agitation, the sample rested for 24 h in a fume hood to allow decantation. The process involved separating the resulting phases and filtering the aqueous phase three times using filter paper before storing the samples for further analysis. The team prepared all aqueous solutions with deionized water.

### 2.2 Synthesis of Carbon Dots

**Figure 1:**
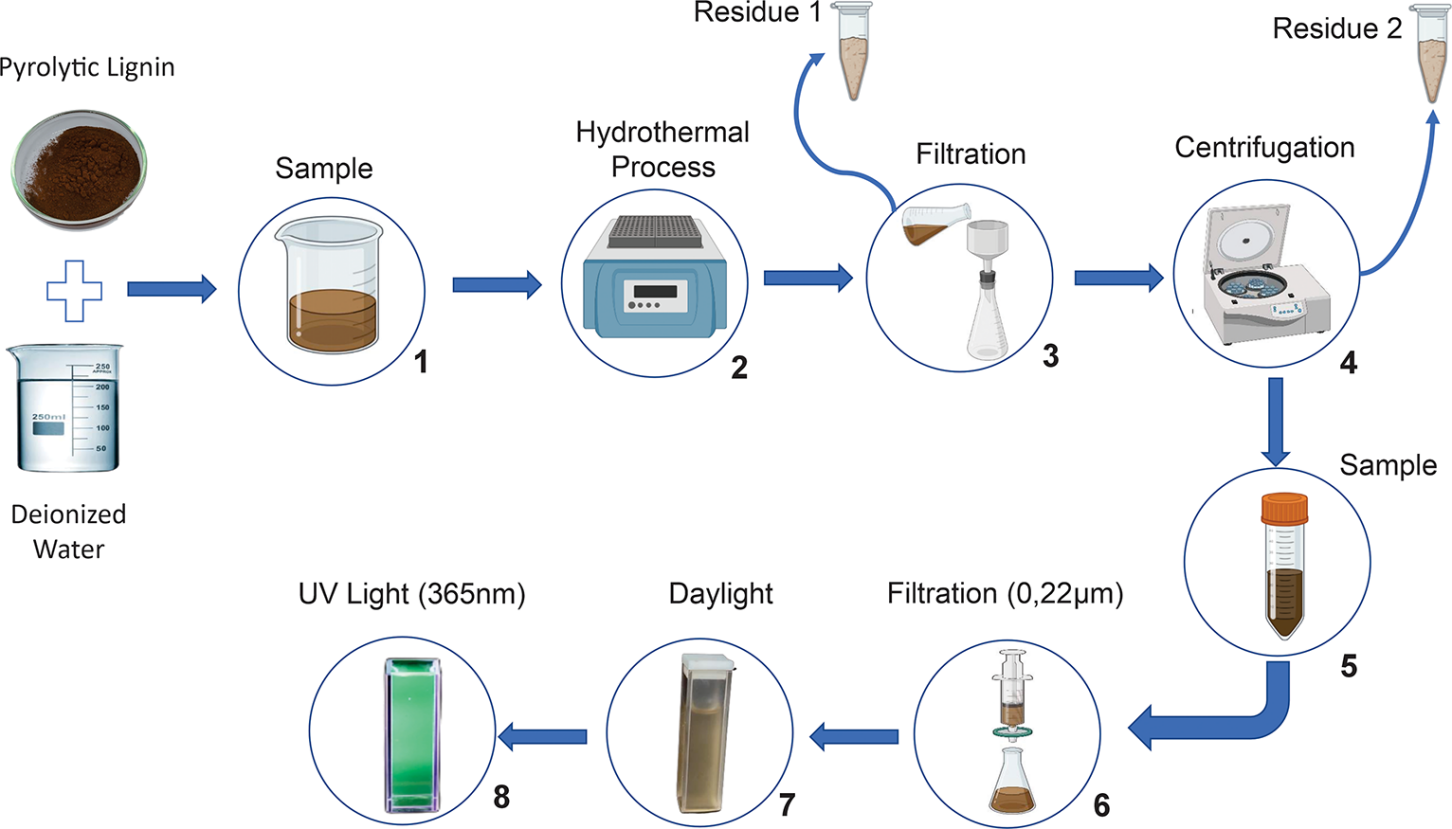
Schematic illustrating the production pathway of pyrolytic lignin-based carbon dots: (1) Mixing pyrolytic lignin with deionized water in a reactor; (2) hydrothermal treatment of the sample; (3) filtration to separate Residue 1; (4) centrifugation to obtain Residue 2 and the liquid sample; (5) collection of the liquid sample; (6) filtration of the sample through a 0.22 μm membrane; (7) observation of the sample under daylight; (8) observation of fluorescence under UV light at 365 nm.

Pyrolytic lignin-based carbon dots were produced as follows: 0.5 g of pyrolytic lignin was dispersed in 30 mL of deionized water and manually stirred to mix the material in water. Then, the dispersion was sealed in a 50 mL Hastelloy steel cylindrical reactor to an aluminum digestion block apparatus (Marconi, Brazil) for hydrothermal treatment at 200 °C for 12 h. After the reaction, the equipment was naturally cooled, and the yellow dispersion obtained was centrifuged in a Eppendorf 5810R centrifuge at 8000 rpm to remove insoluble substances. Subsequently, the material was filtered with a 0.22 μm membrane filter (Kasvi, Brazil) to remove lignin oligomers that did not react during the synthesis of pyrolytic lignin-based carbon dots (Figure 1).

### 2.3 Physical and Chemical Characterization

The surface morphology of pyrolytic lignin-based carbon dots was investigated by transmission electron microscopy (TEM) by using a JEM 1200EX II (Jeol Ltd., Japan) at 200 kV. Fourier transform infrared (FT-IR) spectra were recorded using a Nicolet iS50 (Thermo Fisher Scientific Co.) spectrophotometer with the KBr pellet technique ranging from 400 to 4000 cm^–1^. To determine the particle size and charge of pyrolytic lignin-based carbon dots, a Zetasizer Nano ZS size analyzer (Malvern, U.K.) was used at 25 °C in an aqueous solution of potassium chloride (0.001 mol L^–1^).

### 2.4 Optical Properties of Pyrolytic Lignin-Based Carbon Dots

All fluorescence measurements were recorded in a quartz cell with path length of 10 mm by using an RF-5301 PC (Shimadzu Corp., Japan) fluorescence spectrometer for solutions having absorbance at the wavelength of excitation (λ_ex_). The sample was excited from 300 to 480 nm, in 20 nm increments using slit width of 5 nm. UV/visible absorption spectra (200–700 nm) were recorded by a UV-1800 spectrophotometer (Shimadzu Corp., Japan) using slit width of 2 nm. A pair of quartz cuvettes with a path length of 10 mm was employed for this purpose.

The lifetime analyses were performed using a spectrofluorometer (Fluorolog-3, Horiba Jobin Yvon) with a pulsed excitation nanoLED at 340 nm and a repetition rate of 1.00 MHz.

Fluorescence quantum yield (QY) represents the ratio of photons emitted to photons absorbed by the fluorescent compounds. To determine the quantum yield, a comparative method was employed, utilizing quinine sulfate as a reference compound. Quinine sulfate, dissolved in 0.1 M H_2_SO_4_ solution, was selected as the reference due to its established quantum yield of 54% or 0.54, as reported in the literature.

The quantum yield of fluorescent carbon dots in aqueous solutions was evaluated by comparing the integrated fluorescence intensity (excited at 350 nm) and absorbance (sample with a maximum concentration of 0.1 and a minimum of 0.01 at wavelength excitation) of each sample. This comparative analysis involved calculating the slope (*m*) of the line obtained from plotting the integrated fluorescence intensity against absorbance (0.01–0.1) for both the unknown samples (*m*_X_) and the standard (*m*_R_). Additionally, the refractive index of the medium (water, *n* = 1.3) was considered.

The quantum yield (QY) of the unknown samples was determined using eq 1:
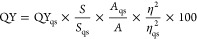
1where, QY represents the quantum yield, *S* the integrated photoluminescence area, *A* the absorbance measured at the excitation wavelength (350 nm), and η the refractive index of the solvent used. The subscript qs is the reference solution, and the values of QY_qs_, η_qs_, and η are 0.54, 1.33, and 1.33, respectively.

The lifetime is the period that excited molecules take to return to the ground state. The radiative decay curve characterizes emission centers in the nanomaterials. The fluorescence lifetime was calculated as follows:
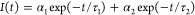
2

Given that *I*(*t*) represents the photoluminescence intensity at time *t*, α is the amplitude of the component, and τ is the lifetime, also known as the time constant.

## 3 Results and Discussion

**Figure 2:**
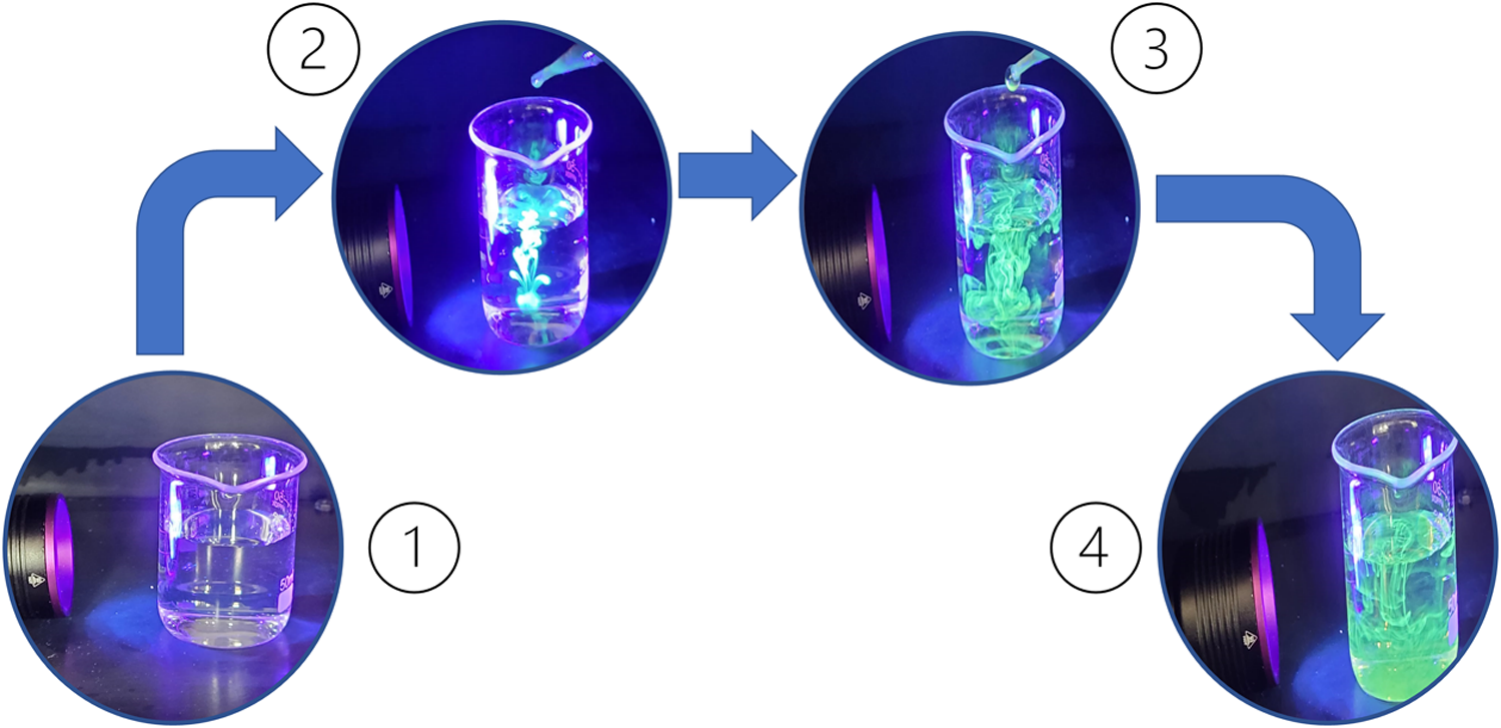
Sequential scheme of dripping pyrolytic lignin-based carbon dots into distilled water under 360 nm UV light: (1) distilled water; (2), (3), and (4) sequential dripping of the nanoparticles into the distilled water.

In the quest for efficient carbon dot production, a cost-effective, straightforward synthesis route is paramount. Herein, we introduce pyrolytic lignin carbon dots synthesized via meticulously controlled thermal pyrolysis of a lignin precursor in deionized water without the use of any other feedstock. The synthesis process involved a one-pot method in which blue/green fluorescent pyrolytic lignin-based carbon dots were easily prepared by heating aqueous dispersion to 200 °C for 12 h (photoluminescence under UV light at 360 nm is visualized in Figure 2).

Pyrolytic lignin, obtained through the phase separation of bio-oil by cold water addition, exhibits hydrophobic aromatic moieties and hydrophilic hydroxyl groups within its side chain.^16^ Pyrolytic lignin agglomeration occurs in an aqueous medium as the temperature increases. The initial agglomerates undergo dehydration, polymerization, and condensation reactions, resulting in the formation of primary aromatic clusters. These reactions occur mainly in the aliphatic regions between different lignin molecules.^26^ With sufficient reaction time and high temperature, the primary aromatic agglomerate undergoes new transformations through aromatization and carbonization processes, leading to the formation of the final pyrolytic lignin-based carbon dots.^27^

**Figure 3:**
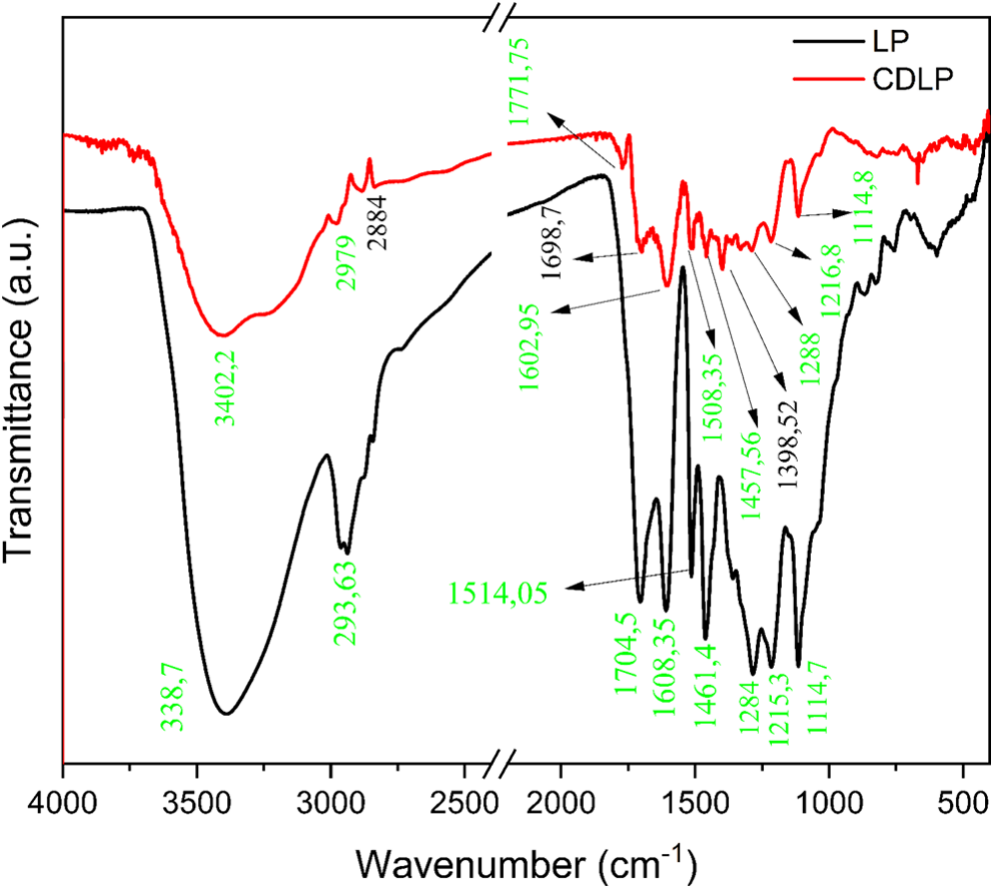
FTIR spectra of pyrolytic lignin (LP) and pyrolytic lignin-based carbon dots (CDLP).

Infrared region analyses were performed to identify the chemical groups present in the pyrolytic lignin and pyrolytic lignin-based carbon dots (Figure 3). No significant changes, other than intensity, were observed related to the mass amount of the product used for the assay. Intense bands around 3400 cm^–1^ were observed as a result of the elongation vibration of the O–H groups. The bands around 2932 and 1460 cm^–1^ were ascribed to asymmetric and symmetric vibrations of C–H bonds of methyl or methylene groups. At 1330 and 1215 cm^–1^, bands correspond to the stretching vibrations of C–O bonds in aromatic structures. The three bands around 1600, 1515, and 1460 cm^–1^ can be associated with the stretching vibrations of C–C bonds in the aromatic chain. Assignments of the bands for the FTIR spectra of pyrolytic lignin and pyrolytic lignin-derived carbon dots are presented in Table S1.

Using ζ-potential analysis, the surface charge of the pyrolytic lignin-based carbon dots was quantified, revealing a value of −10.4 ± 0.14 mV. This observation indicates that the surface is negatively charged, which has significant implications for material interactions and colloidal dynamics. The negative surface charge of the CDPLs is consistent with the presence of oxygen-containing functional groups, particularly carboxyl and hydroxyl groups, which are abundant in the lignin precursor and persist in the final carbon dot structure.^9,15^ These groups tend to deprotonate in aqueous solutions, contributing to the overall negative surface charge.^15^ This characteristic is common among lignin-derived carbon dots and plays a crucial role in their colloidal stability and potential applications. The presence of these functional groups not only influences the surface charge but also contributes to the unique optical properties and potential applications of the CDPLs in various fields, including sensing and bioimaging.^16^

**Figure 4:**
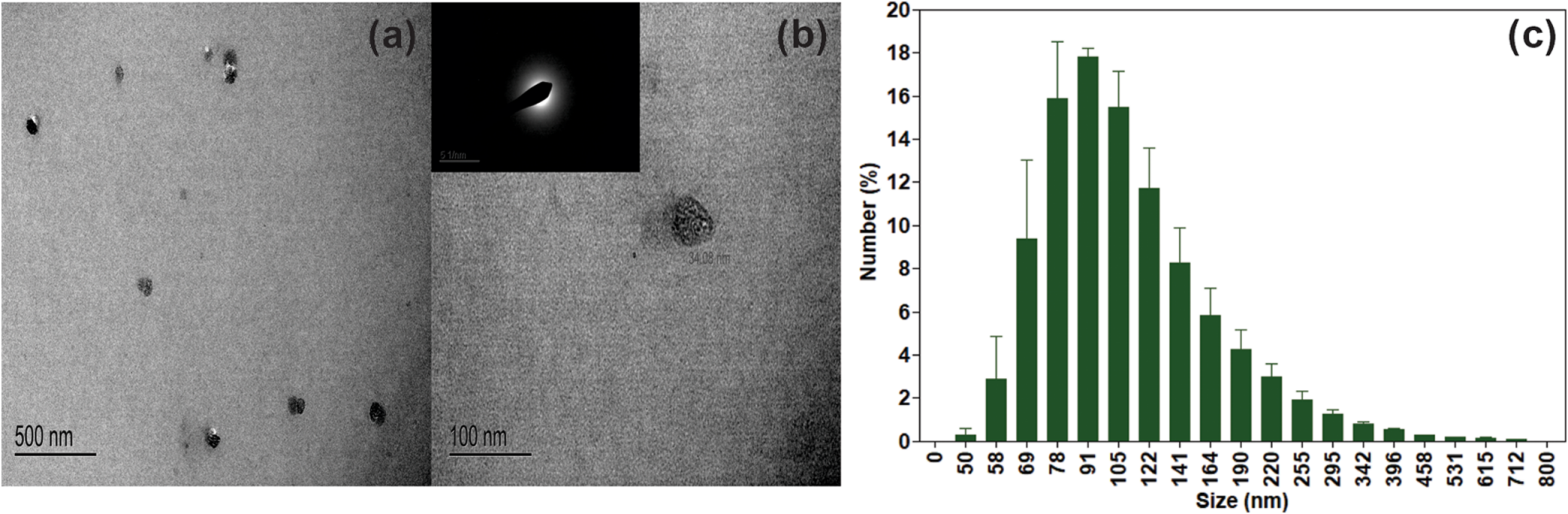
(a) Particle size and morphology of CDPLs measured from TEM images; (b) zoomed region of a single nanoparticle with their SAD diffraction pattern; and (c) hydrodynamic size distribution from the EDL experiment.

The average size of our pyrolytic lignin-based carbon dots (CDPLs) was found to be 34 nm (Figure 4a), which is notably larger than the typical sub-10 nm size range often reported for carbon dots.^15^ This increased size can be attributed to several factors inherent to the pyrolytic lignin precursor and our synthesis process.^14^ The complex and heterogeneous structure of pyrolytic lignin may lead to the formation of larger initial aggregates during hydrothermal treatment.^12,14^ Additionally, the synthesis conditions might result in incomplete breakdown of lignin structures or promote the agglomeration of smaller particles.^3,7^ However, the limited visibility of the particles in the images may have influenced the accuracy of the size statistics, probably due to the more diluted conditions during execution of the technique. Despite this, the particles exhibited a generally spherical morphology and appeared entirely amorphous in the selected diffraction area (SAD top left of Figure 4b), lacking the characteristic rings associated with crystalline materials. While the size of our CDPLs exceeds that of conventional carbon dots, it aligns with observations in other studies of lignin-derived carbon nanomaterials.^15,16^

Furthermore, dynamic light scattering (DLS) was employed to evaluate the particle size, revealing discrepancies in TEM measurements. This technique may encounter challenges with highly photoluminescent particles because a significant decrease in correlation coefficients is observed due to increased absorption of coherent incident light.^19^ Together with the corresponding emission of noncoherent fluorescent light, this can reduce data quality. However, the DLS data indicated a narrow size distribution among the particles, suggesting a well-defined and consistent particle size, although some variation was observed (Figure 4c).

The synthesis yield of pyrolytic lignin-based carbon dots (in triplicate) is illustrated in Figure S2. It was determined in relation to the initial mass of the pyrolytic lignin, its residual fractions (residues 1 and 2), and the product, presenting a value of approximately 64 ± 3.6%. Assuming that the residues are fractions of unconverted lignin oligomers, we inferred that the remainder of the mass was converted to carbon dots. Despite the need for further quantitative improvements, this value indicates a greater conversion of pyrolytic lignin into the product in the suspension than that of solid carbon (hydrothermal carbon). These values are higher than those reported in the literature, such as 0.8–12.06% for alkaline lignin CDs^28^ and 2.39–5.02% for lignin sulfonate fluorescent carbon nanoparticles.^29^ These data indicate the possibility of obtaining carbon dots based on pyrolytic lignin with high conversion values through the hydrothermal method.

The photoluminescence quantum yield (QY) of the pyrolytic lignin-based carbon dots was determined to be 7.9 ± 0.7% at an excitation wavelength of 350 nm, as shown in the standard curve presented in Figure S3. This characteristic can be attributed to several factors. The heterogeneous nature of lignin results in a variety of emissive states, while the presence of nonradiative relaxation pathways due to surface defects and functional groups further impacts the QY.^15,30^ Additionally, the inherent complexity of the carbon dot structure may lead to energy transfer processes that compete with fluorescence emission.^16^ It is noteworthy that our QY value is comparable to or even higher than those reported for other lignin-derived carbon dots, including some with heteroatom doping (Table 1). Studies using only lignin as a precursor have reported QY values as low as 4.62%^28^ and 4.50%,^31^ while even some heteroatom-doped lignin-based carbon dots showed QY values ranging from 7.95%^26^ to 8.10%.^27^ This suggests that our pyrolytic lignin-based carbon dots, without additional dopants, achieve a relatively competitive quantum yield. This QY value is comparable to those reported in the literature for lignin-derived carbon dots synthesized using similar methods, such as hydrothermal carbonization at 200 °C for 12 h^13,31,32^ and solvothermal carbonization at 200 °C for 12 h^33^ with other types of lignin feedstock (Table 1). This QY result demonstrates that pyrolytic lignin-based carbon dots exhibit promising photoluminescent properties, achieving competitive quantum yields without the need for additional dopants or complex synthesis methods.

**Table 1:** Comparison of CDPLs and Carbon Dots Prepared from Other Lignins

yield (QY)	synthesis method	source	reference
1.68%	molecular aggregation	cellulosic enzyme lignin	(34)
7.95%	carbonization and grinding	alkali lignin	(35)
8.10%	carbonization and grinding	alkali lignin	(36)
4.62%	solvothermal carbonization at 200 °C/10 h	alkali lignin	(37)
2.94%	calcination at 250 °C/N^2^	sodium lignosulfonate	(38)
8.23%	hydrothermal carbonization at 200 °C/12 h	cellulosic enzyme Lignin	(32)
7.40%	hydrothermal carbonization at 200 °C/12 h	alkali lignin	(13)
7.00%	hydrothermal carbonization at 200 °C/12 h	alkali lignin	(39)
4.50%	hydrothermal carbonization at 220 °C/12 h	enzymatic hydrolysis lignin	(40)
8–9%	solvothermal carbonization at 200 °C/12 h	alkali lignin	(33)
4.50%	solvothermal carbonization at 180 °C/12 h	alkali lignin	(41)
7.89%	hydrothermal carbonization at 200 °C/12 h	pyrolytic lignin	our study

The production of pyrolytic lignin-based carbon dots using water as a green solvent is particularly relevant in the context of biorefinery systems, where the valorization of lignocellulosic byproducts and residues, such as pyrolytic lignin, is a crucial aspect to ensure sustainability and economic viability of these processes.^16^

**Figure 5:**
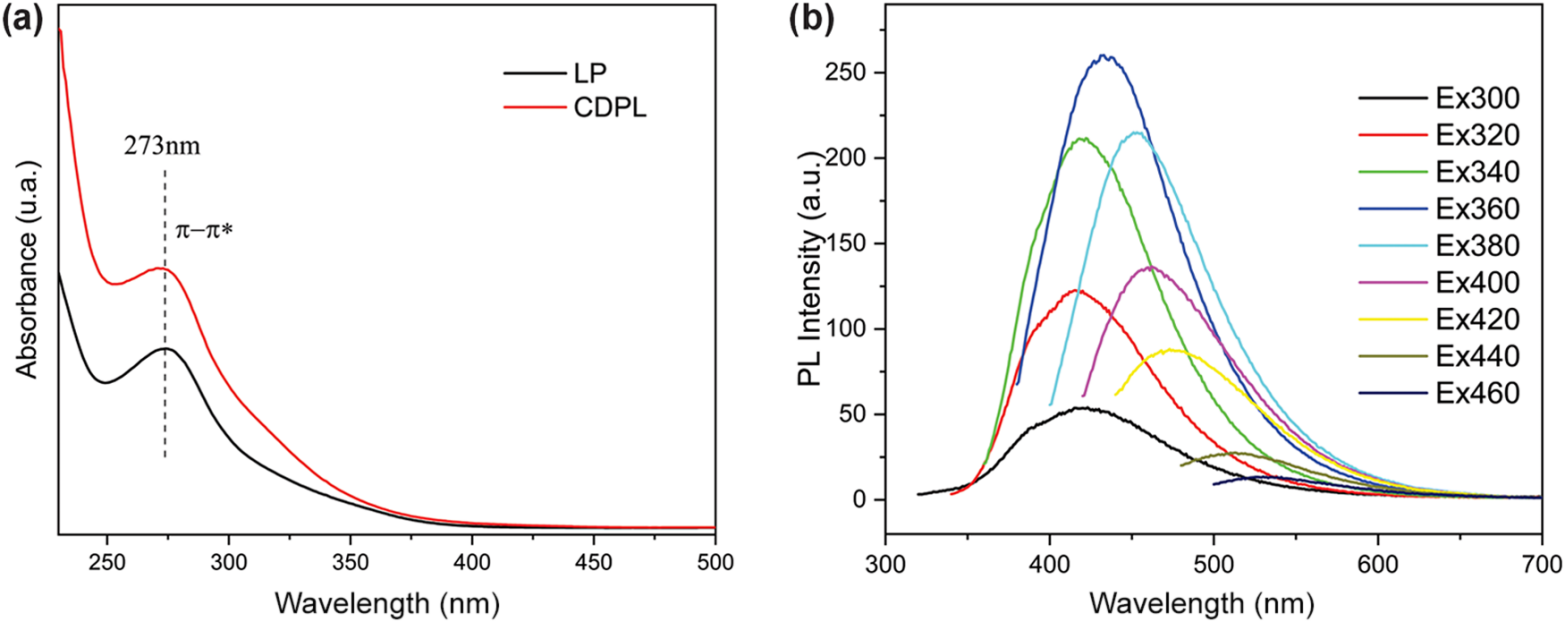
(a) UV–vis spectra of pyrolytic lignin (LP) and pyrolytic lignin-based carbon dots (CDPL) and (b) emission spectra (excitation wavelength gradually increases from 300 to 450 nm) of in aqueous solution.

Regarding the optical characteristics, Figure 5a shows that pyrolytic lignin and pyrolytic lignin-based carbon dots exhibit similar light-absorption patterns in the UV–vis spectrum. A comparative analysis of the FTIR spectra of pyrolytic lignin and the derived carbon dots (CDPLs) reveals both similarities and notable differences in their functional groups and structures. This comparison provides insights into the structural changes occurring during the hydrothermal synthesis process. The broad band around 3400 cm^–1^, present in both pyrolytic lignin and CDPLs, is attributed to the vibrations of the O–H stretching vibrations. Its persistence in CDPLs indicates the retention of hydroxyl groups, which contribute to the hydrophilicity and potential for further functionalization of the carbon dots. The bands at 2932 and 2850 cm^–1^, corresponding to C–H stretching in the methyl and methylene groups, are observed in both materials. However, their relative intensity appears reduced in CDPLs, suggesting partial dehydrogenation during the carbonization process. Notably, the aromatic skeletal vibrations at 1600, 1515, and 1460 cm^–1^ are preserved in CDPLs, although with altered intensities. This indicates the retention of aromatic structures from the lignin precursor, which likely form the core of the carbon dots. The band at 1705 cm^–1^, assigned to C=O stretching in unconjugated ketones and carboxyl groups, is more pronounced in the CDPLs. This suggests an increase in carbonyl-containing functionalities during the hydrothermal treatment, potentially enhancing the surface properties of the carbon dots. The signals at 1330 and 1215 cm^–1^, attributed to C–O stretching in syringyl and guaiacyl units, respectively, are present in both materials but show reduced intensity in CDPLs. This indicates partial breakdown of these lignin subunits during carbon dot formation, while still retaining some of the original lignin structure. The band at 1030 cm^–1^, corresponding to C–O–C stretching in ethers, appears to be more prominent in CDPLs. This could indicate the formation of new ether linkages during the carbonization process, contributing to the cross-linked structure of the carbon dots. Overall, this FTIR analysis demonstrates that while CDPLs retain many of the functional groups present in the pyrolytic lignin precursor, the hydrothermal synthesis process induces significant structural changes. These include partial aromatization, increased carbonyl functionalities, and potential formation of new ether linkages. These structural modifications are crucial in transforming the lignin precursor into fluorescent carbon dots with unique optical and physicochemical properties.

Figure 5b presents the emission spectra of the pyrolytic lignin-based carbon dots in aqueous solution with excitation wavelengths ranging from 300 to 480 nm in 20 nm increments. The main photoluminescence peaks exhibit a blue shift at excitation wavelengths of 300 and 400 nm. The peak intensity initially increases and then decreases, reaching a maximum at an excitation wavelength of approximately 360 nm and an emission wavelength of approximately 440 nm. This phenomenon is commonly observed in carbon-based fluorescent materials, where the emission wavelength and intensity are significantly influenced by the excitation process.^36^ This can be attributed to factors such as the particle size distribution and the presence of multiple functional groups, which lead to distinct emissive states.^42^

Similar to previous studies on different lignin-derived carbon dots,^41−42^ the pyrolytic lignin-based carbon dots exhibit a red shift in the emission band with increasing excitation wavelength, particularly after the 400 nm region. This is mainly due to the relaxation of the polar groups (hydroxyl and carboxyl) on the surface, resulting in a “giant red-edge effect.”^30,45^ The tunability of the emission band allows the optical properties, such as emission color, of pyrolytic lignin-based carbon dots to be tailored, making them suitable for applications requiring control of the spectral response, such as optoelectronic devices.^46^ Furthermore, the red shift of the emission (red-edge effect) can be beneficial for bioimaging applications because light in this spectral range penetrates more deeply into biological tissues.^8,47^

In addition to optoelectronic and sensing applications, pyrolytic lignin-based carbon dots can be utilized as luminescent materials in diverse areas, such as paints, coatings, and markers, because their tunable emission allows customization of their luminescent characteristics.^48^ Therefore, the observed emission band shifts in lignin-based pyrolytic carbon dots open up a wide range of practical applications, from optoelectronic devices and optical sensors to biomedical and energy conversion applications, by exploiting their tunable optical properties.

**Figure 6:**
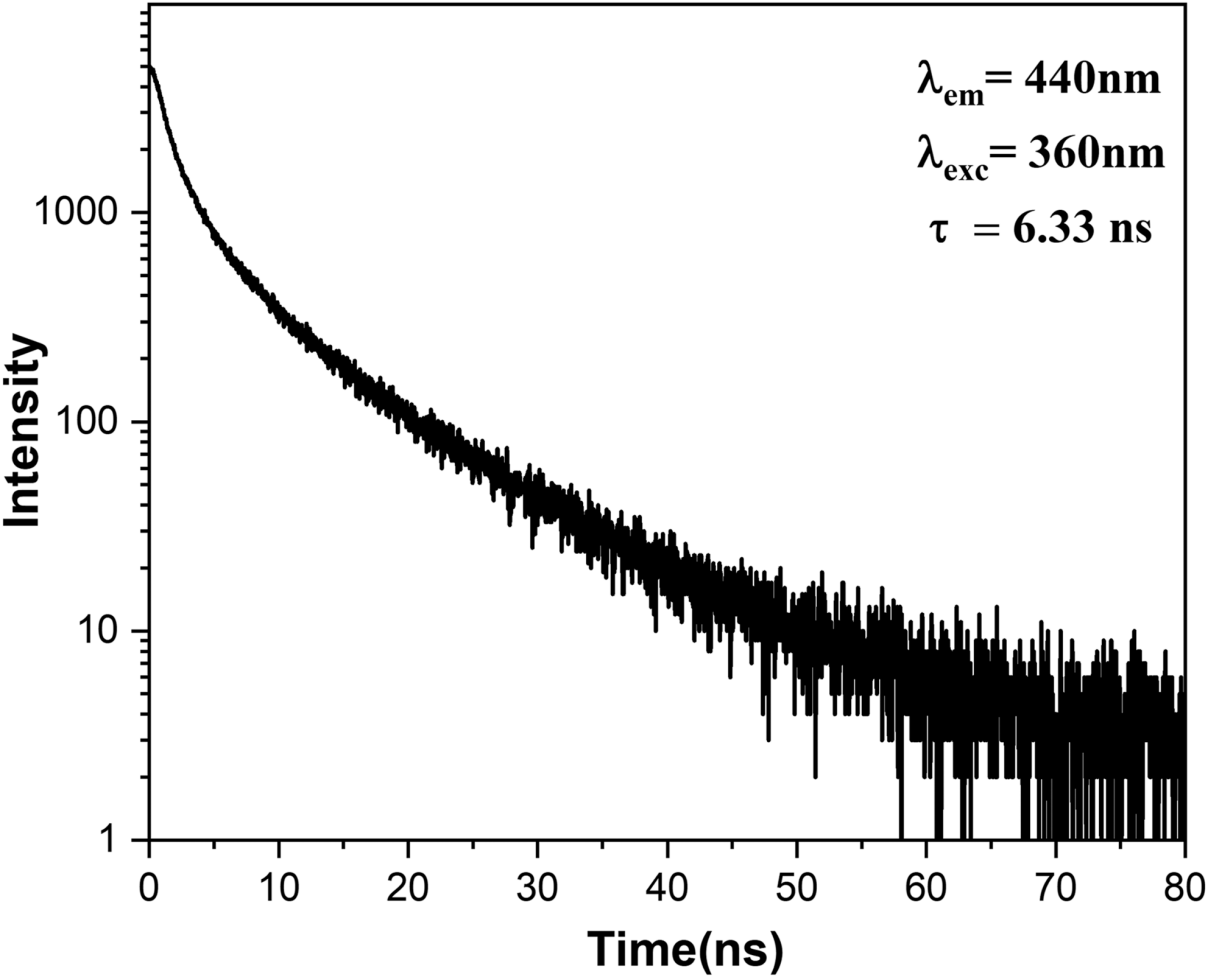
Fluorescence lifetime of pyrolytic lignin-based carbon dots with excitation at 360 nm and emission at 440 nm. The decay curve yields a lifetime (τ) of 6.33 ns.

Figure 6 shows that the carbon dots derived from pyrolytic lignin exhibit a radiative decay lifetime in the nanosecond range with an average lifetime of 6.33 ns, fitted by a biexponential function, as presented in eq 2.

This behavior, typical of carbon dots, is ascribed to recombination processes involving the carbon dots’ intrinsic and surface defect states.^49,50^ Notably, this observed lifetime is longer than the typical sub-5 ns lifetimes reported for many carbon dots, which can be attributed to several factors related to the unique structure and composition of our CDPLs.^24,51^

The complex nature of pyrolytic lignin, with its diverse aromatic structures and oxygen-rich functional groups, likely contributes to a more varied set of emissive states. Additionally, the surface functionalization retained from the pyrolytic lignin precursor may modulate the electronic structure of the CDPLs, potentially slowing down the radiative recombination process.^24,51^

Comparing these values with those in the literature, we found that they are within the lifetime range reported for other lignin-derived carbon dots, albeit on the higher end. For example, carbon dots prepared from alkaline lignin exhibited average lifetimes of 2.79 and 5.19 ns, depending on the synthesis method.^36,52^ However, other papers using alkaline lignin as the raw material but employing different acidic additives obtained shorter lifetimes, ranging from 1.30 to 2.94 ns.^9^ It is worth noting that even longer lifetimes have been reported for some lignin-derived carbon dots, such as the 10.89 ns lifetime observed by Sun et al.^53^ However, it used a lower concentration of lignin (0.05–0.1 g) than other production components, such as ethylenediamine (2.40 g) and citric acid (2.10 g). The present results indicate that the CDs derived from pyrolytic lignin exhibit a radiative decay lifetime consistent with the lifetimes reported in the literature for other lignin-based CDs.

These extended lifetimes can be advantageous for certain applications, such as time-gated bioimaging and sensing, where longer-lived fluorescence can improve signal-to-noise ratios.^24,51^ Our results demonstrate that pyrolytic lignin-based carbon dots exhibit fluorescence lifetimes comparable to or even longer than those of other lignin-derived carbon dots, highlighting their potential for applications requiring extended excited state lifetimes.^24,53^

This study did not directly calculate quantitative green metrics such as E-factor and atom economy, but the approach offers several environmental advantages: using water as the sole solvent minimizes the generation of hazardous waste. In addition, the one-pot hydrothermal synthesis method reduces energy consumption and process complexity compared to multistep procedures. The high yield of carbon dots (approximately 64%) indicates efficient use of the pyrolytic lignin precursor, contributing to waste reduction.

## 4 Conclusions

This study demonstrates a pioneering carbon-based nanoparticle production using pyrolytic lignin from a fast pyrolysis bio-oil. Our research focused on developing a novel and sustainable approach to synthesize carbon dots from this underutilized biomass derivative. The results demonstrated that the proposed method is efficient for the preparation of pyrolytic lignin-based carbon dots. Without the need for additional dopant materials to enhance photoluminescence, the analysis of the luminescence properties revealed that the pyrolytic lignin-based carbon dots exhibited a photoluminescence quantum yield comparable to that reported in the literature. UV–vis and FTIR analyses indicated subtle changes in the absorbance and functional groups present in the feedstock and pyrolytic lignin-based carbon dots. The ζ-potential analysis indicated that the nanoparticles have negative charges. Although the light scattering technique does not have accuracy in determining photoluminescent particle sizes, it is interesting to note the homogeneous distribution of the sample at approximately 10 nm, as measured by TEM. The results of this study indicate the potential for the use of pyrolytic lignin to produce carbon dots using a simple and reproducible production technique. Advances in their characterization and possible application of these pyrolytic lignin-based carbon dots can strongly assist the development of carbon dots from lignocellulosic biomass. This study represents a step toward more sustainable nanomaterial synthesis within the biorefinery concept. We demonstrate the potential for value-added products from lignocellulosic biomass processing streams using pyrolytic lignin, a byproduct of bio-oil production. Future work should focus on comprehensive life cycle assessment and detailed green metrics calculations to fully quantify the environmental impact of this approach.

## Supporting Information Available

Additional information on the assignments of bands for the FTIR spectrum of pyrolytic lignin and carbon dots, quantum yield (in %) of pyrolytic lignin-based carbon dots, and standard curve of quinine sulfate and pyrolytic lignin-based carbon dots. (PDF)
