# Supplementary material for: Pyrolytic Lignin: A Promising Precursor for the Green Synthesis of Fluorescent Carbon Nanoparticles
Source: ACS Omega. 2025 Mar 12;10(11):11054–62. doi: 10.1021/acsomega.4c09764 (PMC11947820; doi:10.1021/acsomega.4c09764)

Pyrolytic Lignin: A Promising Precursor for the Green Synthesis of Fluorescent Carbon Nanoparticles

Rosinaldo Rabelo Aparicio [1,2]; Gabriel Goetten de Lima [3]; Gisele Eliane Perissutti [4], Maiara de Jesus Bassi [5]; Joslaine Jacumazo [6], Marco Antônio Schiavon [7], Lucimara Stolz Roman [1,5], Graciela Ines Bolzon de Muniz [9], Washington Luiz Esteves Magalhães [1,4] Pedro Henrique Gonzalez de Cademartori [1,9]*

[1] Materials Science and Engineering Program (PIPE), Federal University of Paraná, Polytechnic Center, Curitiba, 81531-990, Brazil

[2] Federal Institute Catarinense, 89240-000, São Francisco do Sul, Brazil.

[3] PRISM Research Institute, Technological University of the Shannon: Midlands Midwest, Athlone, N37HD68, Ireland.

[4] Embrapa Florestas, Colombo, 83411-000, Brazil.

[5] Nanostructured Devices Laboratory at Physics Department, Federal University of Paraná, Curitiba, 81531-980, Brazil.

[6] Pharmaceutical Sciences Graduate Program, Federal University of Paraná (UFPR), Curitiba, 81531-990, Brazil.

[7] Materials Chemistry Research Group, Department of Natural Sciences, Federal University of São João del-Rei, São João del-Rei, 36301-160, Brazil.

[9] Forestry Engineering Graduate Program (PPGEF), Federal University of Paraná, Curitiba, 80210-170, Brazil.

*** Corresponding author:** Pedro Henrique Gonzalez de Cademartori, Universidade Federal do Paraná, Curitiba, 80210-170, Brazil. E-mail: pedroc@ufpr.br; Tel.: +55 (41) 996951039.

Table S1 - Assignments of bands for the FTIR spectrum of pyrolytic lignin and carbon dots.

**Wave Number (cm-1)**	**Designation**	**Functional Groups and Structure of Lignin**
3400-3600	O-H	Free -OH
3100-3400	O-H	Associated -OH
2820-2960	C-H	-CH2, -CH3
2920	C-H	Carboxylic -OH
2650-2890	C-H	Methyl Group in –OCH3
1700-1800	C=O	Ketones, non-conjugated carbonyls and esters
1650-1680	C=O	Conjugated p-substituted carbonyl and carboxyl
1500-1600	Aromatic Ring	Benzene rings; C=C stretching in furans
1450-1470	C-H	Asymmetric stretching in -CH2, -CH3
1300-1400	Aromatic Ring	Benzene rings; In-plane C-H deformation
1270-1290	C-O	Guaiacyl
1214-1233	C-O	C-C, C-O, C=O,
1140-1145	C-H	Guaiacyl
1000-1100	C-H, C-O	Aromatic ring; C-O in alcohols and ethers
900-920	C-H	Aromatic ring
850-860	C-H	Out-of-plane C-H at positions 2, 5 and 6 of guaiacyl

Figure S1 - Quantum yield (in %) of pyrolytic lignin-based carbon dots (triplicate).

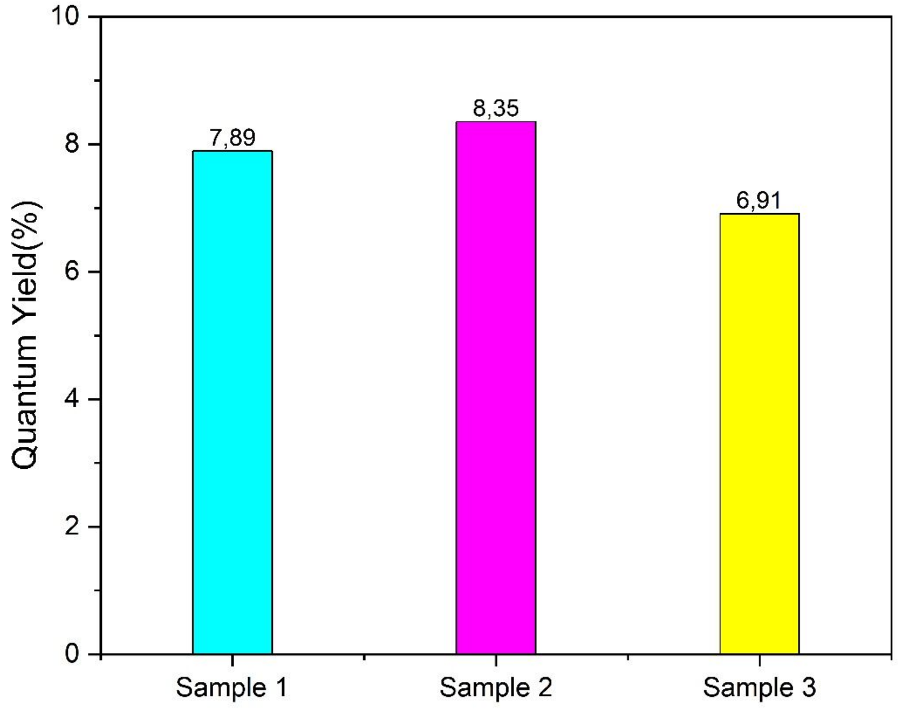

Figure S2 - Standard curve of quinine sulfate and pyrolytic lignin-based carbon dots.

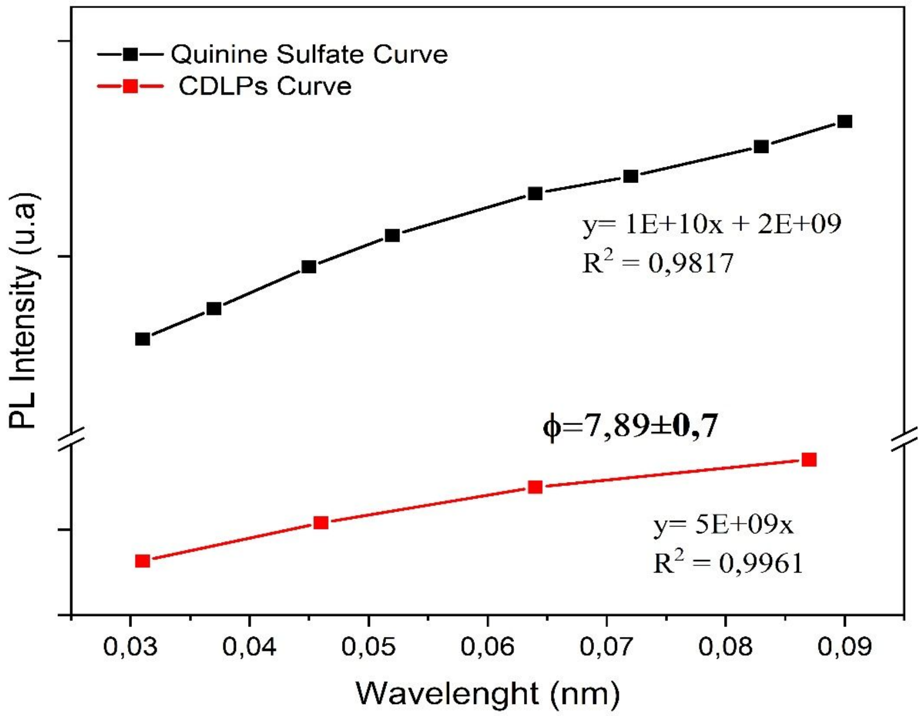

Supplement: Supplementary file 1 — ao4c09764_si_001.pdf [file ao4c09764_si_001.pdf]
